# Proceedings of the inaugural symposium on dengue human challenge studies “Challenging the norm: Accelerating dengue countermeasures through human studies”

**DOI:** 10.1371/journal.pntd.0013889

**Published:** 2026-01-09

**Authors:** Shirin Kalimuddin, Adam T. Waickman, Panisadee Avirutnan, Mohammad Shafiul Alam, Christopher Chiu, Barnaby E. Young, Christopher W. Woods, Petra C. Fay, Sheila K. Pakir, Jo-Anne Manski-Nankervis, Claus Bolte, Andrew Pengilley, Marco Cavaleri, John C.W. Lim, Stephen J. Thomas, Albert I. Ko, Eng Eong Ooi

**Affiliations:** 1 Department of Infectious Diseases, Singapore General Hospital, Singapore, Singapore; 2 Programme in Emerging Infectious Diseases, Duke-NUS Medical School, Singapore, Singapore; 3 Department of Microbiology and Immunology, State University of New York Upstate Medical University, Syracuse, New York, United States of America; 4 Upstate Global Health Institute, State University of New York Upstate Medical University, Syracuse, New York, United States of America; 5 Division of Dengue Hemorrhagic Fever Research and Siriraj Center of Research Excellence in Dengue and Emerging Pathogens, Faculty of Medicine Siriraj Hospital, Mahidol University, Bangkok, Thailand; 6 International Centre for Diarrhoeal Disease Research, Dhaka, Bangladesh; 7 Department of Infectious Disease, Imperial College London, London, United Kingdom; 8 National Centre for Infectious Diseases, Singapore, Singapore; 9 Tan Tock Seng Hospital, Singapore, Singapore; 10 Lee Kong Chian School of Medicine, Nanyang Technological University, Singapore, Singapore; 11 Division of Infectious Diseases, Duke University, Durham, North Carolina, United States of America; 12 Wellcome Trust, London, United Kingdom; 13 Kaye Consulting, Singapore, Singapore; 14 National Healthcare Group Polyclinics, Singapore, Singapore; 15 University Hospital Basel, Basel, Switzerland; 16 Therapeutic Goods Administration, Australian Capital Territory, Canberra, Australia; 17 European Medicines Agency, Amsterdam, the Netherlands; 18 Centre of Regulatory Excellence, Duke-NUS Medical School, Singapore, Singapore; 19 Department of Epidemiology of Microbial Diseases, Yale School of Public Health, New Haven, Connecticut, United States of America; 20 Instituto Gonçalo Moniz, Fundação Oswaldo Cruz, Recife, Brazil; 21 Viral Research and Experimental Medicine Centre, SingHealth Duke-NUS Academic Medical Centre, Singapore, Singapore; 22 Saw Swee Hock School of Public Health, National University of Singapore, Singapore, Singapore; 23 Department of Clinical Translational Research, Singapore General Hospital, Singapore, Singapore; George Washington University School of Medicine and Health Sciences, UNITED STATES OF AMERICA

## Abstract

On 12th June 2025, Duke-NUS Medical School, supported by the Global Dengue and *Aedes*-Transmitted Diseases Consortium (GDAC), hosted a one-day symposium in Singapore titled *“Challenging the norm: Accelerating dengue countermeasures through human challenge studies”.* Comprising sessions on both dengue and other viral infection models, the symposium explored how human challenge studies could address gaps in dengue pathogenesis and immunity to inform vaccine development and application. Keynote speakers highlighted persistent challenges in dengue vaccine development, including incomplete protection across all four dengue viruses, complexities in dengue immunity, and past safety concerns that have influenced regulatory pathways. Case studies from Thailand and Bangladesh illustrated the feasibility and safety of dengue challenge studies in endemic settings. Parallel discussions drew lessons from human infection models of SARS-CoV-2 and other respiratory viruses that are directly relevant to expanding the use of dengue human challenge studies. Sessions on public engagement and regulatory frameworks emphasised the importance of trust, transparent communication, and harmonisation in the design, conduct, and acceptance of challenge studies. Overall, the symposium highlighted that human challenge studies, if implemented thoughtfully, can advance dengue vaccine research, accelerate clinical evaluation and regulatory approval of vaccine candidates, and support evidence-based public health strategies to strengthen global dengue control efforts.

## Introduction

On 12th June 2025, Duke-NUS Medical School, supported by the Global Dengue and *Aedes*-Transmitted Diseases Consortium (GDAC), hosted a one-day symposium titled “Challenging the norm: Accelerating dengue countermeasures through human challenge studies”. Held in Singapore and comprising sessions both on dengue and as well as other viral human infection models, the symposium explored how human challenge studies could be used to fill gaps in our understanding of dengue immunity and pathogenesis—knowledge that is urgently needed both to inform application of current dengue vaccines, as well as accelerate development of next-generation candidates.

The symposium was timely, given the global surge in dengue cases, with its burden projected to increase further in the coming years [1,2]. Vaccines are urgently needed as part of an integrated dengue control effort, yet none of the current available dengue vaccines have demonstrated full protection against symptomatic infection from all four dengue viruses (DENVs) [3,4]. Despite large phase III clinical trials and extended follow-up, data gaps in vaccine safety and efficacy persist. These gaps arose from lower-than-expected attack rates, unpredictability of circulation of each of the four DENVs, and proportionately fewer baseline seronegative compared to seropositive volunteers in endemic regions. There were no cases of DENV-3 and -4 in the phase III clinical trial of TV003 [5], precluding efficacy conclusions for these two DENV types. Uncertainty also remains regarding the lack of efficacy against DENV-3 and -4 in seronegative participants in the TAK003 phase III trial [6]. Furthermore, a patchy understanding of the complexities of the immune response in both protecting against and enhancing severity of dengue further complicates the development and application of dengue vaccines [4]. For example, the role that T cell immunity plays in disease protection and pathogenesis remains understudied, and the immune determinants of protection in tertiary and quaternary dengue are incompletely understood. Rather than requiring additional or larger field trials that further increase the cost of vaccine development, dengue human challenge studies, if thoughtfully designed and implemented, could provide a cost-effective approach to address unanswered questions. Moreover, such models can facilitate early down-selection of vaccine candidates before advancement to costly phase III trials. Overall, dengue human challenge studies have the potential to streamline vaccine development, accelerate regulatory approval and support broader vaccine application.

The goal of this symposium was thus to learn from those who have pioneered human challenge studies globally, both for dengue as well as other viral diseases, and to explore how these models could be implemented safely and ethically Equally important was the call to foster collaboration among scientists, clinicians, regulators, funders, and communicators to refine and improve the models, and advocate for their role in filling knowledge gaps that continue to hinder global dengue control.

The symposium opened with two keynote lectures, followed by a series of scientific talks covering a broad range of topics. These included the history of dengue human challenge studies, case studies of dengue challenge studies in endemic regions, and presentations from investigators who are currently leading SARS-CoV-2 and other respiratory virus challenge studies. The experience gained from conducting such studies, and their value in enabling granular characterisation of clinical, virological and immune responses are broadly applicable and offers valuable guidance to researchers conducting or planning dengue human challenge studies. Notably, Singapore’s COVID-19 human challenge study represents the first such study to be conducted in the country, and provides precedent for establishing other human challenge platforms locally, including for dengue.

A fireside chat then explored public perceptions of human challenge studies and strategies for effective communication with the general population. The program concluded with a panel discussion among regulators examining how challenge studies could be integrated into frameworks for vaccine evaluation and licensure; data generated from dengue human challenge studies are currently not accepted as a basis for vaccine licensure. Encouragingly, the panel signalled an openness to consider inclusion of human challenge data in licensure dossiers, to complement and strengthen data generated from field trials. Each session concluded with a dedicated question-and-answer segment, providing the audience an opportunity to engage directly with speakers and panellists. Discussions were conducted under Chatham House Rules, enabling open and candid exchange of views.

## Session 1

### Keynote 1: Dengue vaccine pain points

Speaker: Stephen J Thomas (SJT), SUNY Upstate Medical University

The symposium’s first session focussed on the key challenges in dengue vaccine development, with an emphasis on defining realistic expectations for efficacy, safety, and use case. SJT opened by acknowledging persisting gaps in the understanding of dengue immunity and pathogenesis. To date, there is no single immune marker which reliably predicts protection against each of the four genetically distinct DENVs. Although a direct causal link has yet to be established between CYD-TDV (Dengvaxia) vaccination and a vaccine-induced immune profile driving more severe breakthrough infections in dengue seronegative recipients [7], the associative link and elevated relative risk of more severe disease in vaccine recipients compared to controls was clear. Dengue vaccine (and other countermeasure) development efforts have all been impacted by the Dengvaxia experience: More stringent long-term demonstration of safety and efficacy is now required by regulators before any dengue vaccine candidate can be considered for licensure.

SJT structured his talk around the concept of a Target Product Profile (TPP) that outlines the ideal characteristics of a vaccine, including target population, efficacy endpoints, delivery method, and tolerability. He walked through major TPP domains and corresponding “pain points” for dengue vaccines:

(i) ***Indication and clinical targets*.** It remains uncertain which clinical outcome should be prioritised as the primary target of protection. Potential outcomes range from prevention of infection, impact on daily functioning, to reduction in severe disease or hospitalisation. This uncertainty complicates the selection of trial endpoints and the formulation of product claims.
(ii) ***Target populations.*** Including vulnerable groups like infants, pregnant women, and immunosuppressed individuals in clinical trials pose ethical and operational challenges. The Zika outbreak in Brazil in 2015 reframed thinking around these populations [8,9], however, vaccine trial inclusion criteria remains narrow.
(iii) ***Formulation and administration.*** Dengue vaccines should ideally be stable, low-volume, and effective after a single dose. Needle-free delivery methods should be considered. Requirements for tetravalent vaccines complicate formulation and introduce challenges of balancing immunogenicity and efficacy.
(iv) ***Efficacy and strain coverage.*** Achieving protection across all four DENVs and their evolving genotypes has proven difficult. As antigenic and immune landscapes continues to shift, questions arise whether immunity from older strains would still confer protection against circulating variants; genotype-specific differences among DENVs of the same type have been shown to affect vaccine-induced neutralisation capacity [10].
(v) ***Safety and tolerability.*** The Dengvaxia experience has cast a long shadow on dengue development. Although no study has shown a conclusive causal mechanistic relationship between vaccination and more severe outcomes, this potential association warrants careful consideration in vaccine trial design and evaluation.

To mitigate these issues, SJT proposed several strategies: using human challenge studies to generate data in smaller, well-characterised cohorts; investing in long-term cohort studies and systematic biobanking to allow retrospective analysis as new observations and molecular technologies emerge; and deploying wearables or mobile platforms to improve real-time symptom capture and reduce participant fatigue. He closed by warning that misinformation and disinformation may derail even the most effective vaccines.

### Keynote 2: Challenges in dengue control

Speaker: Albert I Ko (AIK), Yale School of Public Health

The second keynote focussed on the public health challenges of dengue control, using the example of Brazil during its explosive epidemic in 2024 [11], contextualised through real-world experience with vaccination and vector-control interventions. A key focus was Brazil’s rapid deployment of a reactive dengue vaccination campaign during the 2024 epidemic using the TAK-003 vaccine, targeting adolescents in high-incidence municipalities. AIK presented results from a test-negative case-control study conducted in São Paulo during the campaign, involving over 90,000 adolescents [12]. Although the 2024 outbreak was driven by both DENV-1 and DENV-2, the overall majority of test-positive cases were captured in a period dominated by DENV-1. Vaccine effectiveness against symptomatic dengue, but not stratified by DENV type, was approximately 50% after the first dose, 62% after the second dose, and 68% against hospitalised dengue. Efficacy was maintained over three months, with possible decline in subsequent efficacy, which may have been due to the emergence of DENV-3, or waning immunity in adolescents who received a single vaccine dose.

The session then shifted focus to vector control, specifically Brazil’s experience with Wolbachia (wMel) introgression. AIK presented preliminary data from the ongoing EVITA Dengue trial (NCT04514107), a large cluster-randomised controlled study in Belo Horizonte assessing wMel mosquito population replacement [13]. Despite major challenges such as pandemic-related delays, concerns regarding study team safety, and the 2024 dengue outbreak, high wMel introgression (70%) was achieved in treatment areas. Final results are expected in 2026.

To assess wMel’s performance during outbreaks, retrospective analyses were conducted in Rio de Janeiro using spatial models. wMel replacement exerted a protective effect in the years 2022 and 2023, although this effect appeared to decline during the 2024 outbreak. This decline may reflect confounding factors like changes in risk over time and location, vector density, socioeconomic conditions, or population immunity.

To conclude, AIK stressed the importance of understanding how population susceptibility interacts with intervention impact. Effective vector control would lower the force of infection, increase overall population susceptibility and reduce natural boosting of immunity, raising questions about long-term immunity, waning, and timing of optimal vaccination. He also discussed gaps in our understanding of the disease burden of dengue in the elderly, highlighting that in Brazil, over 50% of dengue deaths in 2024 occurred in people aged over 60, suggesting a possible need to revisit age-based vaccination strategies.

**Key insights from Session 1:**

- Gaps in understanding of dengue pathogenesis and the complexities of dengue immunity have made vaccine development challenging.
- Past safety concerns have reshaped regulatory expectations and slowed dengue vaccine adoption.
- Human challenge studies as well as long-term cohorts could fill data gaps and support faster and safer development of dengue vaccines.
- Brazil’s 2024 epidemic spurred rapid rollout of TAK-003 vaccination with real-world data suggesting effectiveness.
- wMel mosquito population replacement appear to show mixed results in Brazil with between-year variations in effectiveness, but the exact contributory factors remain to be defined.
- Key gaps include how vector control impacts population susceptibility, and how vaccination strategies should be formulated based on local epidemiology.

## Session 2

### History of dengue challenge studies

Speaker: Adam T Waickman (ATW), SUNY Upstate Medical University

This session reviewed the history of dengue human challenge studies over the course of more than a century. ATW opened by emphasising that much of our knowledge on dengue virology and immunology comes from human studies, either observational or experimental, as no animal model accurately recapitulates human dengue disease.

Early 20th century yellow fever challenge studies by Walter Reed generated foundational knowledge in orthoflaviviral infections such as mosquito-borne transmission, incubation periods, and clinical outcomes of direct blood inoculations [14]. Albert Sabin’s subsequent dengue human challenge studies in the 1940s–1950s described homologous versus heterologous immunity, infection kinetics and clinical outcomes of DENV infection [15,16]. Sabin’s studies provided some of the earliest insights into protective immunity and laid the groundwork for modern dengue human infection models.

Currently, two dengue human challenge models are in use, namely, the National Institutes of Health (NIH)-Johns Hopkins-University of Vermont Model, which is an infection model that primarily results in viremia and rash (henceforth referred to as a Controlled Human Infection Model or CHIM) [17–19], and the U.S. Army-SUNY Upstate Medical University-University of Maryland Consortium Model, which is a disease model that recapitulates mildly symptomatic dengue fever [20–23] (henceforth referred to as a Dengue Human Infection model or DHIM). Both models enable detailed sampling, capturing pre-symptomatic viral kinetics and host immune responses, and have also been used to evaluate vaccine [19,24,25] and antiviral candidates [26].

ATW discussed how such models could be used to de-risk vaccine development early, as well as provide insights into the complexities of dengue immunity. He described a recent DHIM study in which volunteers who previously received a tetravalent purified inactivated virus vaccine (PIV) prime followed by a live-attenuated vaccine (LAV) boost were subsequently challenged with a partially attenuated DENV-1 [24]. Unexpectedly, instead of protection, vaccinees showed breakthrough infection with earlier onset RNAemia, increased upregulation of inflammatory pathways, and greater symptom severity compared to unvaccinated controls. This study highlights the value of human challenge studies both in early down-selection of vaccines as well as revealing nuances in dengue immunity.

ATW concluded by emphasising the enduring relevance of human challenge studies. With over a century of use, they remain uniquely positioned to answer fundamental questions about dengue biology and to accelerate evaluation of new countermeasures in a cost-effective, controlled, and ethical manner.

### Controlled human infection model (CHIM) in Thailand

Speaker: Panisadee Avirutnan (PA), Mahidol University, Thailand

PA presented a case study of Thailand’s effort to establish its first dengue human challenge study, highlighting both operational and scientific milestones. Thailand is uniquely placed for dengue human challenge studies as it is endemic for multiple orthoflaviviruses, creating a complex immune landscape that may affect responses to DENV infection and vaccination. Thailand’s CHIM uses the partially-attenuated rDEN2Δ30 strain, pioneered by NIH-Johns Hopkins-University of Vermont. This DENV strain was selected for two main reasons: (1) it replicates poorly in *Aedes aegypti* mosquitoes, which reduces risk of introduction into the community, and (2) is expected to only cause viremia and rash, allowing measurable endpoints with low risk to participants [17].

To establish the model in Thailand, the research team navigated complex ethical and regulatory requirements, supported by existing collaborations between Mahidol and Oxford University. Community engagement, through outreach and social science research, highlighted possible concerns about safety, oversight, and compensation, which directly informed protocol refinement before ethical submission.

The study’s first phase (NCT05476757) enrolled five adult volunteers, who were seronegative for all four DENVs as assessed by plaque reduction neutralisation test (PRNT), but not necessarily naïve to other orthoflaviviruses endemic in Thailand such as Japanese encephalitis virus (JEV) and Zika virus (ZIKV). Unexpectedly, four of the five volunteers developed symptomatic infection consistent with dengue. Post-challenge, capture enzyme linked immunosorbent assay (ELISA) using a mixture of DENV-1–4 virions showed that these four symptomatic volunteers had low IgM:IgG ratios with rapid IgG boosting, a pattern classically associated with secondary dengue infection, whereas the only afebrile volunteer had a higher IgM:IgG ratio and a slower rise in IgG, suggestive of a primary-like DENV infection. These preliminary serologic patterns, together with the known co-circulation of other orthoflaviviruses in Thailand, raise the possibility of immune priming from prior exposure to non-dengue orthoflaviviruses; immunological cross-talk between dengue and related orthoflaviviruses may affect infection outcome [27,28]. Analysis of non-dengue orthoflavivirus antibody responses are ongoing.

This pilot phase confirmed the feasibility of conducting CHIM in Thailand, while also highlighting that immunologic differences in individuals residing in regions endemic for dengue and other orthoflaviviruses such as JEV and ZIKV, compared with individuals from non-endemic regions, may influence challenge outcomes.

### Controlled human infection model (CHIM) in Bangladesh

Speaker: Mohammad Shafiul Alam (MSA), International Centre for Diarrhoeal Disease Research

The development of CHIM in Bangladesh offered another perspective from a dengue-endemic country with rising dengue incidence. Urban centres such as Dhaka and Chittagong experience seasonal outbreaks during the monsoon seasons, with unprecedented large outbreaks in recent years [29]. MSA explained how Bangladesh’s dengue vaccine research, specifically with the NIH-developed TV005 vaccine, began amid this growing dengue burden.

A previous phase II clinical trial conducted in 2016 in Bangladesh demonstrated safety and immunogenicity of TV005 [30], and in collaboration with NIH-Johns Hopkins-University of Vermont, a decision was made to use CHIM to test efficacy of TV005. This marked the first such study in South Asia. The Bangladesh challenge trial was a phase II, randomised, placebo-controlled, double-blind study evaluating the safety and efficacy of TV005 against infection with the partially attenuated rDEN2Δ30 strain (NCT05229354). Given the novelty of human challenge studies in the region, close engagement with regulators and careful risk communication were crucial prior to study initiation. The study enrolled 192 healthy, dengue seronegative adults who were challenged at 6, 12 or 24 months post-vaccination. Symptoms experienced by study participants were generally mild and self-limiting, and included rash, myalgia and fatigue. None of the participants developed a dengue-like illness.

While final efficacy data is awaited at the time of writing of this report, the study demonstrated that CHIM is feasible and safe even in a resource-limited endemic setting. This approach offers a useful tool for accelerating vaccine development timelines and generating locally relevant data.

**Key insights from Session 2:**

- Human challenge studies have refined our understanding dengue transmission, immunity, and the kinetics of viral replication and disease.
- Two dengue human challenge models have currently been established: the NIH–Johns Hopkins-University of Vermont infection model (CHIM) and the U.S Army -SUNY Upstate- University of Maryland disease model (DHIM).
- Implementation in endemic settings requires coordinated engagement with regulatory authorities, ethics bodies, and community stakeholders.
- Challenge outcomes in endemic populations differ from those in non-endemic regions, reflecting the influence of prior orthoflavivirus exposure on infection and immunity. In addition, serology alone may not fully capture the true extent of prior DENV exposure.
- Dengue challenge studies across diverse settings provide unique opportunities to generate context-specific data, deepen understanding of immunity, and de-risk and accelerate vaccine development.

## Session 3

### SARS-CoV-2 human challenge studies: The UK experience

Speaker: Christopher Chiu (CC), Imperial College London

This session shifted focus from dengue to respiratory viruses. Human challenge infection with respiratory viruses, especially influenza viruses, have been more widely applied to evaluate new vaccines than those for dengue. CC opened by highlighting close parallels between the challenges faced in dengue and respiratory virus research: the need for broadly protective vaccines, a better understanding of correlates of protection, and effective models to evaluate interventions in real time. Human challenge studies have a long history in respiratory virus research, dating back to influenza studies in the 1930s [31]. Modern challenge studies have since been refined to explore mucosal immunity, particularly T-cell responses and innate defence mechanisms that are difficult to evaluate in field studies [32–35].

The COVID-19 pandemic thus presented both an opportunity and a challenge to ethically and safely develop a SARS-CoV-2 human infection model to address knowledge gaps during a global health emergency. The UK government’s Vaccine Task Force commissioned a consortium to build this capability in early 2020. Following World Health Organization (WHO) ethical guidance, a high-containment, closely-monitored study was designed to explore early immune responses and viral kinetics, test diagnostics, and potentially evaluate vaccines.

To ensure participant safety, only healthy adults aged 18–30 years, with no evidence of previous SARS-CoV-2 infection or vaccination were eligible for enrolment. Participants were inoculated intranasally with 10 TCID_50_ of Good Manufacturing Practice (GMP)-grade D614G-containing pre-alpha wild-type SARS-CoV-2 virus, and were quarantined in negative-pressure hospital rooms with intensive clinical monitoring and predefined discharge criteria [36]. Of 34 enrolled participants, 53% developed confirmed infection. Upper respiratory tract viral loads peaked early and reached high titres but subsequently declined without initiation of antiviral therapy. Reported symptoms were generally mild and predominantly confined to the upper respiratory tract. Although nearly all infected participants experienced anosmia, this symptom either fully resolved or demonstrated significant improvement by 6 months post-challenge [36].

Since its initiation, the SARS-CoV-2 challenge study has generated valuable data on the kinetics of infection, validating the accuracy and timing of rapid antigen tests, which shaped UK public health policy on testing frequency during the pandemic [36]. Data from the study has also enabled detailed characterisation of early post-infection immune responses [32]. CC highlighted that a unique strength of human challenge studies is the ability to study individuals who have been exposed but do not develop sustained infection. For example, transient SARS-CoV-2 infections were associated with very early nasal mucosa immune cell infiltration, with early CD4+ and CD8+ T cell infiltration associated with abortive infection [32].

These advances have now culminated in the establishment of Mucosal Immunity in Human Coronavirus Challenge (MusiCC) consortium. MusiCC is a global effort funded by the Coalition for Epidemic Preparedness Innovations (CEPI) and the European Union, and was established to support vaccine development for emerging coronaviruses. CC concluded that human challenge studies an important tool not just for pandemic response, but also for pandemic preparedness.

### SARS-CoV-2 human challenge studies: The Singapore experience

Speaker: Barnaby E Young (BEY), National Centre for Infectious Diseases, Singapore

BEY reflected on Singapore’s entry into the field of human challenge studies with the SingCoV study (NCT06654973), a SARS-CoV-2 delta variant challenge study. This study is modelled after the United Kingdom’s SARS-CoV-2 challenge protocol, and is ongoing at the time of writing of this report.

BEY described how earlier attempts to conduct challenge studies in Singapore were hampered by issues such as a lack of suitable quarantine facilities, absence of local experience with human challenge studies, and uncertainties surrounding ethical and regulatory requirements. These barriers have since been removed with the maturation of regulatory and ethical frameworks and the establishment of a purpose-built high-level isolation unit (HLIU) at the National Centre for Infectious Diseases (NCID). The pandemic also shifted public perception of risk; with most Singaporeans previously infected with SARS-CoV-2, volunteers were more willing to participate as they perceived that risk to themselves was low, a finding confirmed through volunteer engagement surveys.

A goal of the SingCoV study has been to demonstrate the feasibility and safety of conducting human challenge studies in Singapore, with a longer-term vision of building a national ecosystem for human challenge research capable of responding to future outbreaks. The research team is now also developing challenge models for dengue and planning infrastructure to support a wide range of future agents. These include creating a volunteer registry, establishing a challenge agent biorepository, and investing in public engagement and ethics research specific to Singaporean contexts. Together, these initiatives aim to establish Singapore as a suitable and sustainable hub for human challenge research.

### Human challenge studies for respiratory viruses

- Speaker: Christopher W Woods (CWW), Duke University

CWW spoke on the use of human challenge studies to accelerate diagnostic innovation, particularly for respiratory viruses. Early U.S. Defense Advanced Research Projects Agency (DARPA)-funded work in the mid-2000s aimed to develop pre-symptomatic diagnostics for deployed military personnel. Human infection models provide the optimal framework to identify infection biomarkers under controlled conditions. Among studies of RSV, influenza, and human rhinovirus, mRNA transcriptomics emerged as the most reliable platform, enabling detection of interferon-related gene signatures within 36 hours of infection [37,38]. These transcriptomic signatures were later validated in real-world cohorts, predicting infection several days before symptom onset [39].

During the COVID-19 pandemic, further studies supported by the U.S. Department of Defense and Biomedical Advanced Research and Development Authority (BARDA) advanced a multi-modal diagnostic platform integrating salivary and capillary blood mRNA, exhaled breath volatiles, and wearable sensor data. In a live influenza H3N2 challenge, transcriptomic profiles closely tracked with viral kinetics, while resting heart rate from wearable sensors were a sensitive early indicator of infection [40].

These diagnostics were developed not only for military use but also to support clinical triage, enable early treatment, and reduce inappropriate antibiotic prescribing by distinguishing viral from bacterial infections. Several platforms have since advanced into commercial application. CWW concluded that human challenge studies have broad applications beyond vaccine development. These include platforms for infectious disease preparedness, personalised health monitoring, and outbreak response, providing insights into host–pathogen dynamics which are difficult to study in natural infection.

### Challenge studies for vaccine development

Speaker: Petra C Fay (PCF), Wellcome Trust, UK

PCF highlighted the strategic role of human challenge studies in vaccine development, particularly for neglected and emerging infectious diseases. Wellcome Trust now embeds human challenge studies within broader vaccine pipelines, prioritising studies that can potentially overcome translational bottlenecks, such as identifying correlates of protection or supporting licensure.

For dengue and Zika, recent efforts included a 2023 workshop and a 2024 funding call supporting five research teams across 16 countries to study host-virus interactions, immunity, and clinical outcomes, with findings informing interventions and potential human challenge studies. Key challenges remain in assay development, especially for virus-specific serology and cell-mediated immunity measurements.

A Program for Appropriate Technology in Health (PATH)-commissioned landscape analysis highlighted global regulatory variability in human challenge study acceptance and recommended capacity building, adaptable frameworks, and expansion of trial sites in endemic regions. Human challenge studies are rarely sufficient for licensure alone, although there have been precedents [41]. They are nonetheless valuable for de-risking development of therapeutic and vaccine by enabling down-selection of candidates with low likelihood of efficacy. Future Wellcome Trust-funded human challenge studies will require clear translational rationale, industry integration, ethical and community engagement, and demonstrable public health impact.

**Key insights from Session 3:**

- Close parallels exist between dengue and respiratory virus research, such as challenges in defining correlates of protection and the need for broadly protective vaccines.
- SARS-CoV-2 human challenge studies have generated valuable information delineating protective host immune responses in exposed individuals.
- Singapore’s regulatory, ethical, and infrastructural frameworks are now well-positioned to support human challenge study capacity building, beginning with SARS-CoV-2 and extending to other high-burden pathogens such as dengue.
- Challenge studies have broad applications beyond vaccine development, including the development of clinically-relevant diagnostics.
- Although unlikely to be sufficient for vaccine licensure alone, challenge studies can provide early efficacy data and support evidence-based down-selection of candidates.

## Session 4

### Fireside chat: Chit-chat at the “Kopitiam”

Panellists: Jo-Anne Manski-Nankervis, LKC School of Medicine; Nicole Lim, Duke-NUS Medical School; Sheila K Pakir, Kaye Consulting

This unscripted panel brought together experts in communication, public policy, and patient engagement to explore the societal and cultural dimensions of human challenge studies. Framed as an informal conversation that typically happens over coffee (*Kopitiam* is a local vernacular reference for coffee shop), the session examined trust, messaging, and the role of public perception in shaping the success of such studies in Singapore.

The panellists stressed that rigorous science alone cannot succeed without public trust and acceptance. They discussed the concept of *pragmatic trust, i.e.,* confidence in institutions based on competence rather than empathy. In Singapore, trust in government and medical authorities is generally high but remains conditional, and can weaken if people feel unheard or poorly informed; community acceptance is therefore not guaranteed. In addition, the heterogeneity of dengue illness and how the public perceives the disease also add a layer of complexity: some see it as a routine and relatively mild illness, while others fear its severe outcomes. The lack of therapeutics, variability in symptoms, and greater risks associated with secondary infection further amplify this complexity. Communicators must explain not only what human challenge studies aim to achieve, but also why these goals necessitate such an approach.

Clear, transparent communication was seen as essential. Informed consent should go beyond legal disclosure to foster genuine understanding and shared purpose. Participants who are aligned with a study’s goals are more likely to maintain trust, even when facing discomfort or adverse events. The panel also highlighted the importance of storytelling and credible messengers. Multimedia tools, such as explainer videos, testimonials, and social media, can humanise science and counter misinformation; these can be designed in partnership with community members. Healthcare professionals, patient advocates, and community leaders may be more trusted voices than government officials, particularly when amplified through grassroots networks.

The discussion concluded that the aim of outreach is not universal persuasion but baseline awareness and legitimacy. The public should understand why human challenge studies are conducted and trust that participants are informed, protected, and respected. As one panellist observed, outreach is not about marketing science, but rather about building durable relationships that can withstand uncertainty.

### Discussion: Regulatory frameworks for filling vaccine data gaps

- Panellists: Andrew Pengilley (Therapeutic Goods Administration, Australia); Claus Bolte (University Hospital Basel); Marco Cavaleri (European Medicines Agency)
- Moderator: John Lim (Centre of Regulatory Excellence, Duke-NUS Medical School)

This session brought together regulatory experts to discuss how human challenge studies (both DHIM and CHIM) might support dengue vaccine approval, in particular filling gaps that phase 3 clinical trials are unable to address. All three panellists agreed that challenge studies offer valuable early-stage insights, especially for DENV types with limited or unknown efficacy data from field trials. In particular, challenge studies were seen as useful for down-selection of vaccine candidates, identifying immune correlates of protection, at least at a minimum threshold given the partially-attenuated phenotype of challenge strains, and generating safety and immunogenicity data in a controlled environment as part of early-phase studies.

However, the regulators were clear that human challenge studies, at least for now, cannot replace large-scale field trials for dengue, particularly for establishing safety and real-world efficacy across diverse populations. Concerns were raised around the use of attenuated viruses, artificial infection routes, and the challenge of replicating natural transmission dynamics. In addition, the role of challenge studies for understanding rare or long-term safety events is currently unclear, which for now can only be captured through large post-marketing or long-term cohort studies. However, the panellists signalled openness to including human challenge data in licensure dossiers, particularly as part of a multi-pronged package including field trials and commitment to post-marketing surveillance.

The panel stressed the importance of designing challenge studies with regulatory relevance in mind from the outset. Protocols should be standardised where possible but adapted to local and pathogen-specific context. The potential for using machine learning tools to fill data gaps on correlates of protection and vaccine efficacy were also raised. Conditional vaccine approvals and population-specific indications could be considered if supported by a robust data package.

Finally, participants called for more international coordination and harmonisation to avoid duplication and improve regulatory clarity. While it is not necessary to conduct human challenge studies in every country, a global agreement on data standards and decision-making frameworks, possibly through World Health Organization (WHO) or other forms of international engagement, would help accelerate vaccine development timelines and ensure that data from challenge models are accepted and trusted across regions.

**Key insights from Session 4:**

- Public understanding and trust, beyond scientific rigour alone, is needed for human challenge studies to be accepted by the lay public.
- Effective communication should prioritise clarity, shared experiences, and trusted messengers over solely legalistic disclosures.
- The primary objective of public outreach in human challenge studies should be to foster baseline understanding and legitimacy, rather than universal persuasion.
- Human challenge studies can generate valuable early-stage data, including immune correlates of protection and enable early down-selection of candidates with low chances of success.
- Regulatory use of human challenge data requires protocols designed with relevance, standardisation, and local context in mind.
- Greater international harmonisation of standards and data acceptance for human challenge studies would accelerate vaccine development and regulatory clarity.

## Conclusion

This inaugural symposium on dengue human challenge studies, which convened a multidisciplinary panel of scientists, clinicians, communicators and regulatory experts, discussed how human challenge studies if applied thoughtfully, could address critical gaps in dengue vaccine research and advance broader dengue control efforts. Their success, however, would require careful study design, regulatory alignment, and clear public communication to safeguard both scientific integrity and societal acceptance. The meeting was unanimous in emphasising the need for continued international collaboration and sharing of best practices to refine and advance challenge model, in support of dengue countermeasure development and global control efforts.

## References

[1] Kalimuddin S, Chia PY, Low JG, Ooi EE. Dengue and severe dengue. Clin Microbiol Rev. 2025;38(4):e0024424. doi: 10.1128/cmr.00244-24 40910631 PMC12697202

[2] Haider N, Hasan MN, Onyango J, Billah M, Khan S, Papakonstantinou D, et al. Global dengue epidemic worsens with record 14 million cases and 9000 deaths reported in 2024. Int J Infect Dis. 2025;158:107940. doi: 10.1016/j.ijid.2025.107940 40449873

[3] Thomas SJ. Is new dengue vaccine efficacy data a relief or cause for concern? NPJ Vaccines. 2023;8(1):55. doi: 10.1038/s41541-023-00658-2 37061527 PMC10105158

[4] Ooi EE, Kalimuddin S. Insights into dengue immunity from vaccine trials. Sci Transl Med. 2023;15(704):eadh3067. doi: 10.1126/scitranslmed.adh3067 37437017

[5] Kallás EG, Cintra MAT, Moreira JA, Patiño EG, Braga PE, Tenório JCV, et al. Live, attenuated, tetravalent butantan-dengue vaccine in children and adults. N Engl J Med. 2024;390(5):397–408. doi: 10.1056/NEJMoa2301790 38294972

[6] Biswal S, Borja-Tabora C, Martinez Vargas L, Velásquez H, Theresa Alera M, Sierra V, et al. Efficacy of a tetravalent dengue vaccine in healthy children aged 4-16 years: a randomised, placebo-controlled, phase 3 trial. Lancet. 2020;395(10234):1423–33. doi: 10.1016/S0140-6736(20)30414-1 32197105

[7] Sridhar S, Luedtke A, Langevin E, Zhu M, Bonaparte M, Machabert T, et al. Effect of dengue serostatus on dengue vaccine safety and efficacy. N Engl J Med. 2018;379(4):327–40. 29897841 10.1056/NEJMoa1800820

[8] Teixeira MG, Costa M da CN, de Oliveira WK, Nunes ML, Rodrigues LC. The epidemic of Zika virus-related microcephaly in brazil: detection, control, etiology, and future scenarios. Am J Public Health. 2016;106(4):601–5. doi: 10.2105/AJPH.2016.303113 26959259 PMC4816003

[9] Costa F, Sarno M, Khouri R, de Paula Freitas B, Siqueira I, Ribeiro GS, et al. Emergence of congenital Zika syndrome: viewpoint from the front lines. Ann Intern Med. 2016;164(10):689–91. doi: 10.7326/M16-0332 26914810 PMC5444536

[10] Martinez DR, Yount B, Nivarthi U, Munt JE, Delacruz MJ, Whitehead SS, et al. Antigenic variation of the dengue virus 2 genotypes impacts the neutralization activity of human antibodies in vaccinees. Cell Rep. 2020;33(1):108226. doi: 10.1016/j.celrep.2020.108226 33027653 PMC7583086

[11] Gurgel-Gonçalves R, Oliveira WKd, Croda J. The greatest dengue epidemic in Brazil: surveillance, prevention, and control. Rev Soc Bras Med Trop. 2024;57:e002032024. doi: 10.1590/0037-8682-0113-2024 39319953 PMC11415067

[12] Ranzani OT, Lazar Neto F, Mareto LK, Brumatti TS, de Oliveira RD, da Silva PV. Effectiveness of the TAK-003 dengue vaccine in adolescents during the 2024 outbreak in São Paulo, Brazil: a test-negative, case-control study. Lancet Infect Dis. 2025. 10.1016/S1473-3099(25)00382-2 40845862

[13] Collins MH, Potter GE, Hitchings MDT, Butler E, Wiles M, Kennedy JK, et al. EVITA Dengue: a cluster-randomized controlled trial to EValuate the efficacy of Wolbachia-InfecTed *Aedes aegypti* mosquitoes in reducing the incidence of Arboviral infection in Brazil. Trials. 2022;23(1):185. doi: 10.1186/s13063-022-05997-4 35236394 PMC8889395

[14] Reed W, Carroll J, Agramonte A, Lazear JW. The etiology of yellow fever: a preliminary note. Public Health Pap Rep. 1900;26:37–53. 19600960 PMC2329228

[15] Sabin AB. Research on dengue during World War II. Am J Trop Med Hyg. 1952;1(1):30–50. 14903434 10.4269/ajtmh.1952.1.30

[16] Snow GE, Haaland B, Ooi EE, Gubler DJ. Review article: research on dengue during World War II revisited. Am J Trop Med Hyg. 2014;91(6):1203–17. doi: 10.4269/ajtmh.14-0132 25311700 PMC4257648

[17] Larsen CP, Whitehead SS, Durbin AP. Dengue human infection models to advance dengue vaccine development. Vaccine. 2015;33(50):7075–82. doi: 10.1016/j.vaccine.2015.09.052 26424605

[18] Hanley JP, Tu HA, Dragon JA, Dickson DM, Rio-Guerra RD, Tighe SW, et al. Immunotranscriptomic profiling the acute and clearance phases of a human challenge dengue virus serotype 2 infection model. Nat Commun. 2021;12(1):3054. doi: 10.1038/s41467-021-22930-6 34031380 PMC8144425

[19] Pierce KK, Durbin AP, Walsh M-CR, Carmolli M, Sabundayo BP, Dickson DM, et al. TV005 dengue vaccine protects against dengue serotypes 2 and 3 in two controlled human infection studies. J Clin Invest. 2024;134(3):e173328. doi: 10.1172/JCI173328 37971871 PMC10836801

[20] Sun W, Eckels KH, Putnak JR, Lyons AG, Thomas SJ, Vaughn DW, et al. Experimental dengue virus challenge of human subjects previously vaccinated with live attenuated tetravalent dengue vaccines. J Infect Dis. 2013;207(5):700–8. doi: 10.1093/infdis/jis744 23225894

[21] Lyons AG. The human dengue challenge experience at the Walter Reed Army Institute of Research. J Infect Dis. 2014;209 Suppl 2:S49-55. doi: 10.1093/infdis/jiu174 24872396

[22] Endy TP, Wang D, Polhemus ME, Jarman RG, Jasper LE, Gromowski G, et al. A phase 1, open-label assessment of a dengue virus-1 live virus human challenge strain. J Infect Dis. 2021;223(2):258–67. doi: 10.1093/infdis/jiaa351 32572470

[23] Waickman AT, Newell K, Lu JQ, Fang H, Waldran M, Gebo C, et al. Low-dose dengue virus 3 human challenge model: a phase 1 open-label study. Nat Microbiol. 2024;9(5):1356–67. doi: 10.1038/s41564-024-01668-z 38561497

[24] Lyke KE, Chua JV, Koren M, Friberg H, Gromowski GD, Rapaka RR, et al. Efficacy and immunogenicity following dengue virus-1 human challenge after a tetravalent prime-boost dengue vaccine regimen: an open-label, phase 1 trial. Lancet Infect Dis. 2024;24(8):896–908. doi: 10.1016/S1473-3099(24)00100-2 38679035

[25] Kirkpatrick BD, Whitehead SS, Pierce KK, Tibery CM, Grier PL, Hynes NA, et al. The live attenuated dengue vaccine TV003 elicits complete protection against dengue in a human challenge model. Sci Transl Med. 2016;8(330):330ra36. doi: 10.1126/scitranslmed.aaf1517 27089205

[26] Durbin AP, Van Wesenbeeck L, Pierce KK, Herrera-Taracena G, Ebone L, Buelens A, et al. Daily mosnodenvir as dengue prophylaxis in a controlled human infection model. N Engl J Med. 2025;393(21):2107–18. doi: 10.1056/NEJMoa2500179 41297006 PMC12662413

[27] Zambrana JV, Hasund CM, Aogo RA, Bos S, Arguello S, Gonzalez K, et al. Primary exposure to Zika virus is linked with increased risk of symptomatic dengue virus infection with serotypes 2, 3, and 4, but not 1. Sci Transl Med. 2024;16(749):eadn2199. doi: 10.1126/scitranslmed.adn2199 38809964 PMC11927040

[28] Malhotra S, Gupta BP, Uranw S, Mantri CK, Rathore APS, St John AL. Dengue disease severity in humans is augmented by waning Japanese encephalitis virus immunity. Sci Transl Med. 2025;17(814):eads9572. doi: 10.1126/scitranslmed.ads9572 40901927

[29] Sharif N, Sharif N, Khan A, Dey SK. The epidemiologic and clinical characteristics of the 2023 dengue outbreak in Bangladesh. Open Forum Infect Dis. 2024;11(2). 10.1093/ofid/ofae066 PMC10883285 38390460

[30] Walsh M-CR, Alam MS, Pierce KK, Carmolli M, Alam M, Dickson DM, et al. Safety and durable immunogenicity of the TV005 tetravalent dengue vaccine, across serotypes and age groups, in dengue-endemic Bangladesh: a randomised, controlled trial. Lancet Infect Dis. 2024;24(2):150–60. doi: 10.1016/S1473-3099(23)00520-0 37776876 PMC11267251

[31] Killingley B, Enstone J, Booy R, Hayward A, Oxford J, Ferguson N, et al. Potential role of human challenge studies for investigation of influenza transmission. Lancet Infect Dis. 2011;11(11):879–86. doi: 10.1016/S1473-3099(11)70142-6 21798808

[32] Lindeboom RGH, Worlock KB, Dratva LM, Yoshida M, Scobie D, Wagstaffe HR, et al. Human SARS-CoV-2 challenge uncovers local and systemic response dynamics. Nature. 2024;631(8019):189–98. doi: 10.1038/s41586-024-07575-x 38898278 PMC11222146

[33] Rapeport G, Smith E, Gilbert A, Catchpole A, McShane H, Chiu C. SARS-CoV-2 human challenge studies—establishing the model during an evolving pandemic. N Engl J Med. 2021;385(11):961–4. doi: 10.1056/NEJMp2106970 34289273

[34] Habibi MS, Thwaites RS, Chang M, Jozwik A, Paras A, Kirsebom F, et al. Neutrophilic inflammation in the respiratory mucosa predisposes to RSV infection. Science. 2020;370(6513):eaba9301. doi: 10.1126/science.aba9301 33033192 PMC7613218

[35] Paterson S, Kar S, Ung SK, Gardener Z, Bergstrom E, Ascough S, et al. Innate-like gene expression of lung-resident memory CD8+ T cells during experimental human influenza: a clinical study. Am J Respir Crit Care Med. 2021;204(7):826–41. doi: 10.1164/rccm.202103-0620OC 34256007 PMC8528532

[36] Killingley B, Mann AJ, Kalinova M, Boyers A, Goonawardane N, Zhou J, et al. Safety, tolerability and viral kinetics during SARS-CoV-2 human challenge in young adults. Nat Med. 2022;28(5):1031–41. doi: 10.1038/s41591-022-01780-9 35361992

[37] Zaas AK, Chen M, Varkey J, Veldman T, Hero AO 3rd, Lucas J, et al. Gene expression signatures diagnose influenza and other symptomatic respiratory viral infections in humans. Cell Host Microbe. 2009;6(3):207–17. doi: 10.1016/j.chom.2009.07.006 19664979 PMC2852511

[38] Barton AJ, Hill J, Pollard AJ, Blohmke CJ. Transcriptomics in human challenge models. Front Immunol. 2017;8:1839. doi: 10.3389/fimmu.2017.01839 29326715 PMC5741696

[39] Tsalik EL, Henao R, Nichols M, Burke T, Ko ER, McClain MT, et al. Host gene expression classifiers diagnose acute respiratory illness etiology. Sci Transl Med. 2016;8(322):322ra11. doi: 10.1126/scitranslmed.aad6873 26791949 PMC4905578

[40] Woods CW, McClain MT, Chen M, Zaas AK, Nicholson BP, Varkey J, et al. A host transcriptional signature for presymptomatic detection of infection in humans exposed to influenza H1N1 or H3N2. PLoS One. 2013;8(1):e52198. doi: 10.1371/journal.pone.0052198 23326326 PMC3541408

[41] Chen WH, Cohen MB, Kirkpatrick BD, Brady RC, Galloway D, Gurwith M, et al. Single-dose live oral cholera vaccine CVD 103-HgR protects against human experimental infection with *Vibrio cholerae* O1 El Tor. Clin Infect Dis. 2016;62(11):1329–35. doi: 10.1093/cid/ciw145 27001804 PMC4872293

